# Mineral Springs of Virginia

**Published:** 1838

**Authors:** 


### Art. VIII.—Mineral Springs of Virginia.

*Notices of the Blue Sulphur, and Hot Springs, of the State of Virginia.* By one of the Editors.

For several years past, the Mineral Springs of the Ancient Dominion, have been constantly gaining in their attractions, on the long and far-famed waters of the Empire State. To the people of the Valley of the Mississippi, *almost* the exclusive readers of our Backwoods' Quarterly, they are, from their comparative proximity, and their mountain locality, of peculiar interest. The access to them by the village of Guyandotte, is by no means difficult; as a good *summer* turnpike, stretching over an alpine region, peculiarly grand and picturesque, extends from the Ohio river to their very midst. The luxury and real value of a mountain atmosphere, in summer, to those who inhabit a hot and sultry plain, can scarcely be overrated. We presume, therefore, that a few pages designed to make some of these watering places more extensively known, will not be unacceptable to any, into whose hands our Journal may chance to fall. We confess, however, that our materials for this purpose are not as racy and copious, as the fountains of which we speak. For the sketches of scenery and accommodations we may give, we are indebted to one of our fellow citizens,* who visited several of them during the past year.

### The Blue Sulphur.

"This spring is the most westerly, and the youngest of the celebrated 'sulphurous sisters,' as a letter writer has called these springs. It is in Greenbriar county, on the great road leading across the mountains, 12 miles west of Lewisburg, and 21 west of the White Sulphur. The spring is situated in a beautiful little valley, on the bank of a very small stream, and is hemmed in by high hills and mountains on every side. The buildings for the accommodation of the visitors are extensive and conveniently arranged; and the lawn which stretches from them to the spring, is pleasant, and embellished with some beautiful sugar maples.

"Near the spring is a bath-house, which is kept in excellent condition. It is supplied with the sulphur water, either hot or cold.

"The quantity of water discharged by this spring is not very great. At the bottom of the spring lies a slight deposite, of a bluish purple hue. The water is not unpleasant to the taste."

We are indebted to a respectable physician of Western Virginia, for a few paragraphs on the effects of this water.

It is diuretic—moves the bowels without pain—excites the liver to increased secretion—determines an action on the skin—and regulates the uterine functions, in amenorrhœa, dysmenorrhœa, and leuccorrhœa. It sometimes seems to exhilerate the general system. It has done much good in dyspepsia and diseases of the liver. Bathing in it, has relieved rheumatism. In gleet it has been found decidedly beneficial. Applied to the skin it has cured herpetic eruptions. Some cases of incipient scrophula have yielded to its use. In one case at least, known to our informant, it not only righted up a constitution deeply impaired by intemperance, but seemed to destroy the desire for ardent spirits! A notable exhibition of its powers.

### The Hot Springs.

We derive from the traveller already quoted, the following notice of these springs.

"They are situated directly on the great road from Guyandotte

---

*B. Drake, Esq.

to Staunton, in one of the narrow vallies with which the mountains abound. The scenery around them is sublime. For three or four days past, we have had frequent, but not copious showers. The weather is now serene again—the sky of a light blue—the air dry and elastic—the sun bright, but not intensely hot. The thermometer seldom rises, during the summer, higher than 85 degrees; and in the valley where I am now writing, there is rarely a foggy morning, though the mountain tops are often hid by a thin mist, which, as the sun rises, passes off in the form of clouds, presenting a beautiful tracery of vapour, assuming a thousand fantastic shapes, and an endless variety of hues. Life in the mountains is truly delightful: it seems to produce a buoyancy of the physical and intellectual man, that is not often felt in a champaign country. The towering pine-covered peaks—the bare and beetling cliffs—the quiet little valleys, through which the rivulets wend their way—the rapid alternations of sunshine and shower, and the varying and gorgeous clouds, now coursing, now reposing on the mountain tops, while stage after stage comes dashing down the winding turnpike, with the pealing notes of the horn echoed from cliff to cliff,—these are magical in their combined effect, and kindle up the imagination and the feelings, to a point of bright and joyous enthusiasm.

"There are five separate and comfortable frame houses, with dressing rooms and fire places, in which the baths are taken. The boiler or sweat bath, for gentlemen, has a uniform temperature of 106 degrees; while that of the ladies' boiler is 103. The gentlemen's hot spout bath, in which the water falls in a copious stream from a height of four feet above the surface of the pool, has a temperature of 106 degrees; the ladies' hot spout bath being the same. The two pleasure baths, one for either sex, are of the temperature of 98 degrees. The pool, in all of these baths, is large enough to afford the exercise of swimming; the water being about four and a half feet in depth. The spout baths are more frequently used than any others. The water, when it first falls upon the body, produces a sensation of scalding; but in an instant, more pleasurable emotions ensue. The usual period for remaining in the spout bath, is from twelve to fifteen minutes. The boiler bath is so constructed, as to exclude, in a great measure, the external air. In taking this bath, the individual usually remains in the water until perspiration commences; he is then wrapped in blankets, and laid upon a cot in one of the ten or twelve little rooms, connected with the bathing-house, where he is left for 30, 50, or 60 minutes, until the perspiration becomes most profuse, and often penetrates blankets and mattrass, and even trickles to the floor. The covering is then gradually removed, and the body cooled by degrees. Under this course there is but little danger of taking cold; and the effect, in chronic disease of long standing, is, in many cases, truly wonderful.

"The attention of Dr. Goode, the intelligent, moral and hospitable proprietor, has been chiefly turned upon the bathing department.

"The long celebrated Hot Springs, is to the rheumatic, the gouty, the lame and the halt, what the Red Sulphur Spring is to the consumptive—the fabled fountain of rejuvenescence. Out of the one hundred persons now here, it would not be an easy matter to form a cotillion, unless cripples, crutches and canes were tolerated in the dance. However, if the visiters cannot gracefully 'trip it on the light fantastic toe,' they would at least make capital soldiers; for in case of an attack, it would be quite impossible for them to run away."

By an application to Dr. Goode, we lately obtained a considerable mass of interesting facts, concerning the curative effects of the Hot Springs, of which we shall avail ourselves, in the composition of the remainder of this brief notice. In this letter the Doctor says, "These waters, when taken internally, are *antacid*, mildly *aperient*, *powerfully diuretic* and *diaphoretic;* and when used externally, they *equalize* an *unbalanced circulation—promote glandular secretion—excite the action of the absoıbent vessels—and allay pain.*"

Dr. Goode's letter encloses the certificates of 10 or 12 gentlemen, from different parts of the United States, setting forth the benefits they had derived from visiting these springs.

One of them had a chronic inflammation of the tonsils, and upper extremity of the larynx. He took 42 sweat baths, sojourned at the spring 57 days, gained 22 lbs. of flesh, and left them with a voice nearly restored, and his general health much improved.

Another had derangements of the abdominal functions, with enlarged spleen, bulimia, and an uncontrollable diarrhœa, of two or three years' standing; the consequence of cholera, bilious fever, and ague, in a southern climate. In a few hours after using the hot bath, he had a bilious dejection, which had not occurred before for eight months; in four days, his diarrhœa ceased, and the alvine discharges became natural, his spleen was soon reduced to half its preternatural dimensions, his complexion improved, and in short all his symptoms gave way. This patient was a medical gentleman.

A third, after suffering for years with chronic rheumatism, in nearly all the joints of his body, and being reduced to a state of great decripitude, was entirely cured, by 45 bathings, between the months of July and September.

The effects of these waters on another rheumatic, were still more remarkable. He was attacked with the disease in an acute form, in January, and, in the following July, was so much debilitated as to require two assistants, to put him in his carriage. He remained at the springs 63 days, and took the bath 60 times, during which he gained in weight 60 lbs. The cure was perfect and permanent.

A gentleman afflicted for months with a neuralgic disease of one of his arms, till it wasted away to an alarming degree, and his general health and strength declined, was taken to the Hot Springs, and employed the bath and blankets 40 times in the course of two months, when he was perfectly restored.

Another, afflicted with gall stones, attended with gastrodynia and jaundice, was *relieved* several times, by visiting the White Sulphur, when he determined to try the Hot Springs. The spout bath, in eight or ten days, effected a perfect cure.

A lady who had been in bad health for more than 15 years, and whose liver was so much enlarged as to project palpably beyond her ribs, visited the springs. While there, she used some aperient medicine, and was bled, but resorted to the baths, diligently, for about a month, when the enlargement of the organ was entirely removed, and she returned home in, comparatively, good health.

A gentleman, by what was regarded as a functional disease of the liver, was greatly reduced in strength, the tone of his stomach was impaired, and his bowels so torpid, that for more than a year, he had had no alvine evacuations without the aid of enemata. His weight had fallen from *two hundred and eighty*, to *one hundred*

*and thirty* pounds! After using the bath for five or six weeks, he left the springs, greatly improved. During the next 12 months, he gained 100 lbs. in weight, and at the end of that time, considered his health as perfectly reinstated.

Another gentleman, after repeated attacks of dysentery, was left with an obstinate mucous diarrhœa, which continued for more than three years. He tried the White and Red Sulphur Springs with but partial success; when he determined on visiting the Hot. The spout bath was directed over his liver and back, with immediate and surprising relief. In a few days the diarrhœa ceased, and the pains in his abdomen, with which he had long been afflicted, both day and night, entirely left him.

These abstracts of documents, in which we have confidence, will show our readers, that the Hot Springs of Virginia are really and rapidly curative, in a variety of rheumatic and abdominal diseases. These are the affections in which the hot and warm springs of Europe have long been celebrated. It is to be regretted, that in the extensive valley of the Mississippi, we have no hot springs, except those of the distant Arkansas, which situated far in the south, are not attractive in summer. We are believers in the superior efficacy of natural to artificial hot bathing; and suppose that the internal use of the water of hot springs cannot fail, in general, to be productive of much benefit.

We have said nothing of the composition of the waters of which we have spoken, as the analyses hitherto made of them, are said not to be of the most accurate character. Professor Rogers, of the University of Virginia, the Geologist of the State, has, however, subjected them to a rigorous examination, and when his report shall be published, we propose to make it the subject of another article. D.

